# Efficient Screening of CRISPR/Cas9-Induced Events in *Drosophila* Using a Co-CRISPR Strategy

**DOI:** 10.1534/g3.116.036723

**Published:** 2016-10-28

**Authors:** Nanci S. Kane, Mehul Vora, Krishna J. Varre, Richard W. Padgett

**Affiliations:** Waksman Institute, Department of Molecular Biology and Biochemistry, Cancer Institute of New Jersey, Rutgers University, Piscataway, New Jersey 08854-8020

**Keywords:** CRISPR, co-CRISPR, co-conversion, *Drosophila*, Cas9

## Abstract

Genome editing using the Clustered Regularly Interspaced Short Palindromic Repeats (CRISPR) and associated nuclease (Cas9) enables specific genetic modifications, including deletions, insertions, and substitutions in numerous organisms, such as the fruit fly *Drosophila melanogaster*. One challenge of the CRISPR/Cas9 system can be the laborious and time-consuming screening required to find CRISPR-induced modifications due to a lack of an obvious phenotype and low frequency after editing. Here we apply the successful co-CRISPR technique in *Drosophila* to simultaneously target a gene of interest and a marker gene, *ebony*, which is a recessive gene that produces dark body color and has the further advantage of not being a commonly used transgenic marker. We found that *Drosophila* broods containing higher numbers of CRISPR-induced *ebony* mutations (“jackpot” lines) are significantly enriched for indel events in a separate gene of interest, while broods with few or no *ebony* offspring showed few mutations in the gene of interest. Using two different PAM sites in our gene of interest, we report that ∼61% (52–70%) of flies from the *ebony*-enriched broods had an indel in DNA near either PAM site. Furthermore, this marker mutation system may be useful in detecting the less frequent homology-directed repair events, all of which occurred in the *ebony*-enriched broods. By focusing on the broods with a significant number of ebony flies, successful identification of CRISPR-induced events is much faster and more efficient. The co-CRISPR technique we present significantly improves the screening efficiency in identification of genome-editing events in *Drosophila*.

The CRISPR/Cas9 system has revolutionized molecular genetics by allowing precise, efficient genome editing in many types of organisms (Cowan 2016; Harrison *et al.* 2014; Hsu *et al.* 2013; Mohr *et al.* 2016; Sander and Joung 2014; Strong and Musunuru 2016; Tasan *et al.* 2016; Xiong *et al.* 2016). Originally discovered as part of the immune system of bacteria and archaea, Cas9 endonuclease cleaves highly specific DNA targets, determined by the guide RNA (gRNA) sequence (Cong *et al.* 2013; Jinek *et al.* 2012; Mali *et al.* 2013; Qi *et al.* 2013). Cleavage by Cas9 endonuclease causes double-strand breaks in DNA, which stimulate DNA repair. Repair can be accomplished by nonhomologous end joining (NHEJ) or, in the presence of a template or donor DNA, by homology-directed repair (HDR) (Dickinson *et al.* 2015; Harrison *et al.* 2014; Norris *et al.* 2015). NHEJ repair can be imprecise, resulting in deletions, substitutions, small insertions, and translocations (indels) that are useful for disrupting gene function. HDR requires a donor DNA template, which can be modified to contain point mutations or DNA insertions (such as GFP) so that the endogenous gene can be tagged for further studies.

Various approaches to genome editing using CRISPR/Cas9 in *Drosophila* have been reported. *In vitro* transcribed RNAs for both Cas9 and gRNAs can be coinjected into wild-type embryos (Bassett *et al.* 2013); *in vitro* transcribed gRNAs (Bassett *et al.* 2013; Gratz *et al.* 2014) or gRNA expression plasmids (Gratz *et al.* 2014; Port *et al.* 2014; Ren *et al.* 2013; Sebo *et al.* 2014) can be injected into transgenic *Drosophila* embryos expressing Cas9, or the gRNAs can be stably integrated by *P*-element or with ϕC31 transformation and then crossed to Cas9 transgenic flies (Chen *et al.* 2015; Kondo and Ueda 2013; Port *et al.* 2014). As in other species, the frequency of NHEJ mutagenesis is much higher than HDR in *Drosophila* (Harrison *et al.* 2014; Mohr *et al.* 2016). However, the frequency of both types of repair events is lower than is ideal, and many techniques are being developed to increase the frequency of CRISPR-induced mutations (Beumer *et al.* 2013; Chu *et al.* 2015; Maruyama *et al.* 2015). Lower than ideal frequencies, coupled with the fact that many desired CRISPR-induced mutations do not produce visible phenotypes, require more laborious screening by PCR, T7 endonuclease assays (Hwang *et al.* 2013), and/or sequencing.

Since the frequency of CRISPR-induced mutations is low, we sought to improve the screening process in *Drosophila* to help identify broods that were more likely to contain CRISPR-induced events. Several studies have shown that multiple CRISPR events can occur in a single cell (Cong *et al.* 2013; Wang *et al.* 2013), enabling the development of a co-CRISPR or coconversion technique in *Caenorhabditis elegans* (Arribere *et al.* 2014; Kim *et al.* 2014; Ward 2015). These three approaches relied on creating a visible phenotype in a marker gene while simultaneously targeting a gene of interest. The high frequency lines with the co-CRISPR or coconversion event have been termed “jackpot” lines, as they often also contained a CRISPR-induced mutation at the gene of interest (Paix *et al.* 2015). In this study, we developed a co-CRISPR strategy in *Drosophila* targeting the recessive gene, *ebony* (*e*). The advantages of this approach in *Drosophila* are that *e* mutations are not lethal, are easy to score, and are not part of the common transgenic fly strategies. Crossing the injected offspring with *e* flies and identifying their *e* broods allows the quick identification of jackpot broods. We show that these jackpot broods have a higher incidence of mutations in the gene of interest, thus allowing one to focus on the most important broods. In our experiments, embryos with transgenically integrated nos-Cas9 nuclease (Port *et al.* 2014) were injected with a mix of gRNAs targeting *e* and *lambik* (*lbk)*, with or without a repair template containing a FLAG-HA tag for HDR insertion in *lbk*, depending on the experiment. Their progeny were then screened for ebony body color and analyzed for mutations.

We show that an *e* co-CRISPR in *Drosophila* provides a valuable, time-saving technique when screening for CRISPR-induced mutations. We demonstrate that flies with CRISPR events in *e* are significantly enriched for CRISPR events in a different gene of interest, with a dramatic increase in detection of NHEJ mutagenesis events and in the rarer HDR knock-in events in these broods. In contrast, no mutations were found in *lbk* in which *e* was not mutated. Use of this technique to identify jackpot lines can reduce the number of flies that need to be screened for CRISPR-induced mutations.

## Materials and Methods

### Fly stocks

All fly stocks were maintained at 25°. The following fly stocks were used: y[1] M{w[+mC]=nos-Cas9.P}ZH-2A w[*] [Bloomington *Drosophila* Stock Center (BDSC) no. 54591]; *w[1118]/Dp(1;Y)y[+]*; *CyO/Bl[1]*; *TM2/TM6B*, *Tb[1]* (BDSC no. 3704); *y^1^ w^67c23^*; *In(2LR)Gla*, *wg^Gla-1^/SM6a*, *CyO* (K. McKim, Rutgers University); w[*]; *L[2] Pin[1]/CyO*, *P{w[+mC]=GAL4-Kr.C}DC3*, *P{w[+mC]=UAS-GFP.S65T}DC7* (BDSC no. 5194); w1118; *Df(2R)ED2487*, *P{3′.RS5+3.3′}ED2487/*SM6a (BDSC no. 29661); and *w^a^* *N^fa-g^*; *Df(2R)Jp8*, w+*/*CyO (BDSC no. 3520).

### Plasmid construction

**Figure 1:**
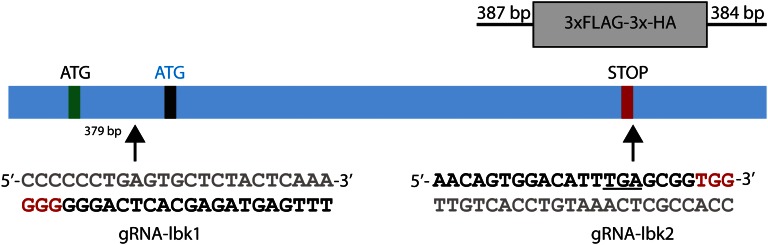
Schematic representation of the *lbk* gene, positions of gRNAs, and knock-in cassette. We selected two guides: one at the N-terminus between two putative start sites on the antisense strand (bottom), gRNA-lbk1, and the other overlapping the termination codon on the sense strand (top), gRNA-lbk2. The gRNA sequences are shown in black with PAM sites in red, while gray indicates the complementary sequence. The underline in gRNA-lbk2 indicates the termination codon. The knock-in cassette consisted of a 225 bp 3xFLAG-3XHA flanked by 387 and 384 bp *lbk* homology arms. The length of genomic *lbk* from the first putative ATG start codon to the TGA stop codon is 4629 bp.

Genomic *lbk* target sites were identified at http://tools.flycrispr.molbio.wisc.edu/targetFinder/ (Gratz *et al.* 2014). Genomic *lbk* is 4629 bp from the first putative start codon to the stop codon. The first *lbk* genomic target, lbk-1, is near the N-terminus 379 bp downstream of the first putative start and 51 bp upstream of the second putative start, and the second target, lbk-2, intersects the stop at the C-terminus (Figure 1). Prior to final selection of *lbk* guides, genomic PCR and sequencing of nos-Cas9 flies was performed to check for polymorphisms in the target regions. Guides targeting *e* (gRNA-e; our plasmid ID 83380, deposited at Addgene) and *lbk* (gRNA-lbk1 and gRNA-lbk2) were inserted in vector *pCFD3: U6:3-gRNA* (Addgene no. 49410) and were constructed as described (Port *et al.* 2014; see http://www.crisprflydesign.org/wp-content/uploads/2014/05/Cloning-with-pCFD3.pdf). The dsDNA repair template for HDR was created by synthesizing a gene block (gB) (Integrated DNA Technologies, Inc.), PCR amplifying it, and inserting it into TOPO vector (TOPO-gB-lbk-FLAG-HA; Thermo Fisher Scientific, Inc.). The gB was 1 kb long and included 387 bp of the *lbk* coding region upstream of the *lbk* stop, deletion of the *lbk* stop codon, mutation of the PAM site, an insertion of a 3xFLAG-3XHA tag (228 bp), insertion of a stop codon, followed by another 384 bp of genomic sequence downstream of the *lbk* stop. All guides were verified by sequencing. The sequences of the oligonucleotides used to construct each gRNA expression plasmid and HDR repair template are shown in Supplemental Material, Table S1 and Figure S1.

### Microinjection

nos-Cas9 embryos were microinjected by BestGene Inc. with two different injection mixes: (1) three plasmid gRNAs in pCFD3 expression vector: gRNA-e, gRNA-lbk1, and gRNA-lbk2 at concentrations of 150 ng/µl each in order to generate targeted mutations by NHEJ, hereafter referred to as the mutagenesis experiments; (2) gRNA-e in pCFD3(100 ng/µl), gRNA-lbk2 in pCFD3 (100 ng/µl), and TOPO-gB-lbk-FLAG-HA (500 ng/µl), in order to generate the HDR knock-in of a FLAG-HA tag at the end of *lbk*, hereafter referred to as the knock-in experiments.

### Genetics and screening

**Figure 2:**
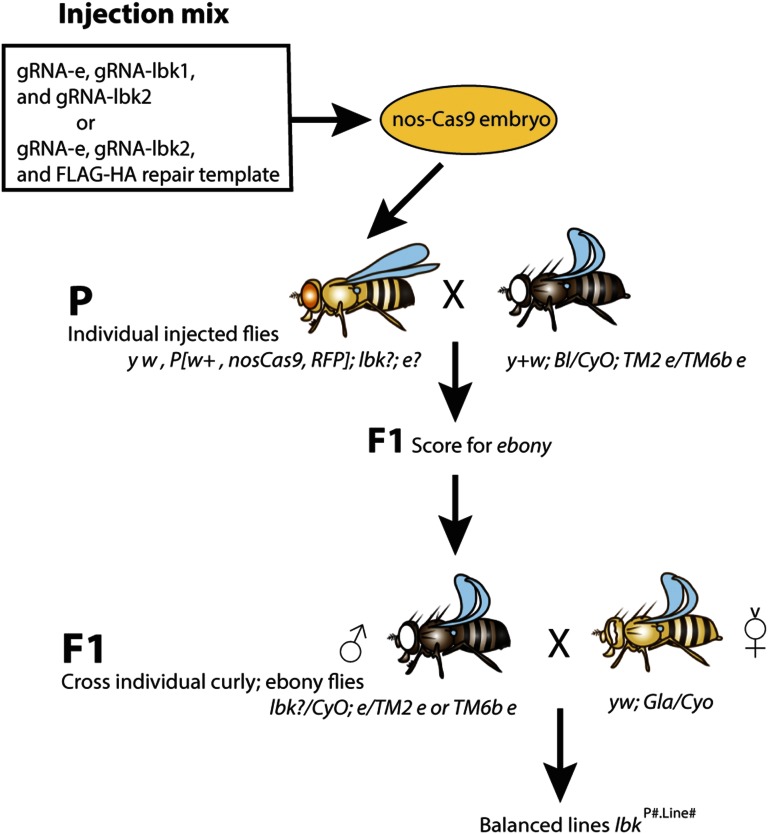
Genetic scheme for screening with co-CRISPR. Transgenic nos-Cas9 embryos were injected with a mix of either gRNA-e, gRNA-lbk1, and gRNA-lbk2 expression plasmids or with gRNA-e, gRNA-lbk2, and a knock-in repair template containing a FLAG-HA tag. Injected individuals were crossed to the ebony double balancer (P cross). F1 offspring were scored for *e* and *e+*. Individual F1 curly, ebony flies were crossed to a second chromosome balancer (F1 cross) to generate second chromosome *lbk* balanced lines. The F1 cross shown is with a single curly, ebony male. After the *e* broods are identified, following *e* is unnecessary. Homozygotes were then analyzed for mutations in *lbk*. Fly images were created on Genotype Builder Photoshop file S5 (Roote and Prokop 2013).

Individual injected adults, whose germ lines contained possible CRISPR events in *lbk* (chromosome 2) or *e* (chromosome 3), were crossed to the double balancer stock *w[1118]/Dp(1;Y)y[+]*; *CyO/Bl[1]*; *TM2*, *e/TM6B*, *e*, *Tb[1]* (BDSC no. 3704) in the Parental (P) cross. P crosses were numbered P♂# or P**☿**# to indicate the gender of the injected fly (for example, P**☿**73 was injected fly number 73, virgin female.) Each F1 brood was scored for ebony or wild-type body color, and the percentage of ebony was calculated. To attempt to capture *lbk* mutations that were segregating from *e*, we established lines from ∼6 F1 flies from many of the broods (Figure 2). These balanced lines generated from single F1 flies were named *lbk^P#.line#^* (for example, *lbk^73.1^* and *lbk^73.2^* are separate but sibling lines from the same injected parent, number 73). Homozygous lethal lines were crossed to w[*]; *L[2] Pin[1]/CyO*, *P{w[+mC]=GAL4-Kr.C}DC3*, *P{w[+mC]=UAS-GFP.S65T}DC7* (BDSC no. 5194) and rebalanced over *CyO*, *Kr-GFP* so that homozygous embryos or larvae could be selected for genomic DNA preps. After scoring the F1 for *e* and *e+* and setting up balanced lines from individual F1 flies, one does not have to follow *e* as it is only predictive of which brood to focus on.

### Genomic DNA preps and identification of mutations

Genomic DNA preps were performed on a single homozygous male from each homozygous viable line or a homozygous L2 larva from homozygous lethal lines, as described (http://vosshall.rockefeller.edu/protocols/SingleFlyGenomic.pdf). nos-Cas9 flies were used for the wild-type control. In brief, one fly or larva was crushed in 50 µl squishing buffer (SB: 10 mM Tris-HCl pH 8, 1 mM EDTA, 25 mM NaCl) plus freshly added Proteinase K (20 µl of 10 mg/ml Proteinase K stock solution in 1× PBS added to 1 ml SB). Lysates were first incubated at 37° for 30 min, then heated at 95° for 3 min to inactivate the Proteinase K, and finally spun down briefly and the supernatant pipetted to a new tube. Using 0.5 μl of the genomic DNA lysate as a template, DNA sequences surrounding the target sites lbk-1 and lbk-2 were amplified by PCR for 32–34 cycles in a 20 μl reaction with 2× PCR Super Master Mix (BioTool B46015). Then, 2 µl of PCR products were analyzed by agarose gel electrophoresis to confirm presence of correct band size, and the remaining PCR products were cleaned with either Zymo Clean & Concentrator-25 (Zymo Research D4033) or ExoSAP-IT (Affymetrix no. 78200) and were sequenced with one of the forward primers (primer sequences are shown in Table S1).

### Data availability

The gRNA-e plasmid is available from Addgene (ID 83380) and the *lbk* gRNAs (gRNA-lbk1 and gRNA-lbk2) are available upon request. Representative fly strains from these experiments are available upon request. All data necessary for confirming the conclusions in this article are represented in the figures and tables.

## Results

### Rate of CRISPR-induced mutations in the e marker gene

Given the success of co-CRISPR in *C. elegans*, we sought to develop a similar system in *Drosophila* to allow for rapid screening of desired mutations. We considered many dominant and recessive mutations and settled upon *e* to develop it as a co-CRISPR marker. In order to exploit using *e* in these experiments, we needed to identify an *e* target site that worked at a reasonable frequency. In some of the early studies developing CRISPR techniques, a target site was identified in *e* (Port *et al.* 2014). We injected gRNA-e at a concentration of either 150 ng/µl for the mutagenesis experiments (mix of gRNA-e, gRNA-lbk1, and gRNA-lbk2) or 100 ng/µl for the knock-in experiments (mix of gRNA-e, gRNA-lbk2, and repair template) (Figure 2). In our experiments with this *e* guide, we calculated both the percentage of injected individuals that produced ebony offspring, and also the percentage of *e* within each individual F1 brood. Sixty-eight fertile P animals were obtained from the mutagenesis experiments, and 43 from the knock-in experiments. Individual P were crossed to *e* double balancer flies to uncover *e* and maintain lethal mutations in *lbk*. All F1 offspring were scored for ebony body color; not all P produced ebony offspring. We observed *e* F1 in 42 (62%) of the mutagenesis broods and 17 (40%) of the knock-in broods (Table 1A and Table 2A), possibly due to the difference in injection concentrations. Within each brood, the percentage of ebony offspring was calculated and ranged from 0 to 100% for the mutagenesis experiments (Table 1B and Table S2) and 2–81% for the knock-in experiments (Table 2B). These broods were classified into one of the following groups based on the percentage of flies with ebony body color: 0%, 1–50% low, and >50% jackpot. In our experiments, we arbitrarily name lines from broods that produced 51% or higher *e* F1 as jackpot lines.

**Table 1:** Germline transmission rates for mutations in *e* and *lbk*

(A) No. of Fertile P	No. (%) of Broods from P Crosses Without *e*	No. (%) of Broods from P Crosses with *e*	No. of F1 Lines Sequenced
68	26 (38)	42 (62)	120
nos-Cas9 embryos were injected with a mix of gRNA-e, gRNA-lbk1, and gRNA-lbk2. *P* indicates the number of injected, fertile flies used in Parental crosses. The rate of *e* mutations: of the 68 P, 38% produced no ebony offspring, and 62% produced ebony offspring, with individual broods ranging from 1 to 100% ebony. 120 F1-derived balanced lines were sequenced. 235 sequences of the two *lbk* genomic regions were analyzed.			

(B) % *ebony* in F1 Broods	No. of Lines Sequenced for lbk-1	No. (%) of Lines with lbk-1 Mutations	No. of Lines Sequenced for lbk-2	No. (%) of Lines with lbk-2 Mutations	No. (%) of Lines with Both lbk-1 and lbk-2 Mutations
0	24	0 (0)	24	0 (0)	0 (0)
1–50	54	12 (22)	51	25 (49)	10 (20)
>50	42	22 (52)	40	28 (70)	14 (36)

nos-Cas9 embryos were injected with a mix of gRNA-e, gRNA-lbk1, and gRNA-lbk2. Each F1 brood is grouped by percentage of ebony: 0, 1–50% low, and >50% jackpot. Performing a two-tailed Fisher’s Exact test (2 × 2 contingency table), we show that for lbk-1, there is a strong statistical significance when comparing the 0% ebony group to either the 1–50% ebony (*P* < 0.05) or the >50% ebony group (*P* < 0.0001) for presence of mutations at lbk-1. Additionally, there is a significant increase in the number of mutations at lbk-1 when comparing the 1–50% to >50% ebony group (*P* < 0.005). Similarly, for lbk-2, we show that there is a strong statistical significance when comparing the 0% ebony group to either the 1–50% (*P* < 0.0001) or the >50% ebony group (*P* < 0.0001) for presence of mutations at lbk-2.

**Table 2:** Frequency of HDR knock-in events

(A) No. of Fertile P	No. (%) of Broods from P Crosses Without *e*	No. (%) of Broods from P Crosses with *e*	No. of F1 Lines Analyzed
43	26 (61)	17 (40)	72
nos-Cas9 embryos were injected with a mix of gRNA-e, gRNA-lbk2, and a repair template. *P* indicates the number of injected, fertile flies used in Parental crosses. The rate of *e* mutations: of the 43 P, 61% produced no ebony offspring, and 40% produced ebony offspring, with individual broods ranging from 2 to 81% ebony. 72 crosses were set up from ebony F1 individuals, and the resulting balanced lines were analyzed by PCR for an HDR insert.			

(B)% *ebony* in F1 Broods	No. of Lines Analyzed	No. (%) of Lines with Knock-In
1–50	43	1 (2)
>50	29	3 (10)
Top two jackpot broods	11	2 (18)

nos-Cas9 embryos were injected with a mix of gRNA-e, gRNA-lbk2, and a repair template. Each F1 brood is grouped by percentage of ebony: 1–50, >50% jackpot, and the top two jackpot broods (a subset of the >50% jackpot broods). We first tested lines from the two best jackpot broods and found an HDR knock-in rate of 18%. Our overall insertion rate in jackpot lines was 10% and only 2% for low ebony broods, suggesting enrichment between percentage of ebony in F1 broods and HDR knock-in events.

### Generation of balanced F1 Lines

Individual F1 flies (all *e* except controls) were crossed to second chromosome balancer flies in order to generate lines balanced for *lbk*, with a goal of six individual lines per brood (Figure 2). If the co-CRISPR events in *lbk* also occurred at a high rate, six lines from each brood would give us a high probability of preserving the putative mutation. For the mutagenesis experiments, over 135 balanced lines were set up, 120 of which were further analyzed by sequencing, including 96 lines from 32 *e* broods and 24 lines from four *e*+ broods for controls (Table 1). For the knock-in experiments, 72 *e*-derived lines were established and analyzed for knock-in events (Table 2A). Once a brood is scored for the presence of ebony body color and balanced lines are generated, it is not necessary to follow ebony in future generations.

### Correlation between CRISPR events in the comarker e and gene of interest, lbk

For the mutagenesis experiments using gRNAs to generate targeted modifications, our data show that lines from jackpot broods are significantly enriched for CRISPR events in lbk-1 and lbk-2 compared to the low *e* and the *e+* lines. In these experiments, we included two gRNAs from the *lbk* gene, one near the 5′ end of the gene and one near the termination codon, in order to test two targets with our new co-CRISPR methodology. This approach could also allow for the rarer event of a deletion between the two gRNAs, a 4.2 kb fragment of the *lbk* gene, as well as mutate either end of the coding region (Figure 1). Deletions of this size have been previously generated, but they are less common (Chen *et al.* 2014; Cong *et al.* 2013; Kondo and Ueda 2013). While we expected the deletion to be homozygous lethal, we nonetheless tested all lines for indels by sequencing.

**Figure 3:**
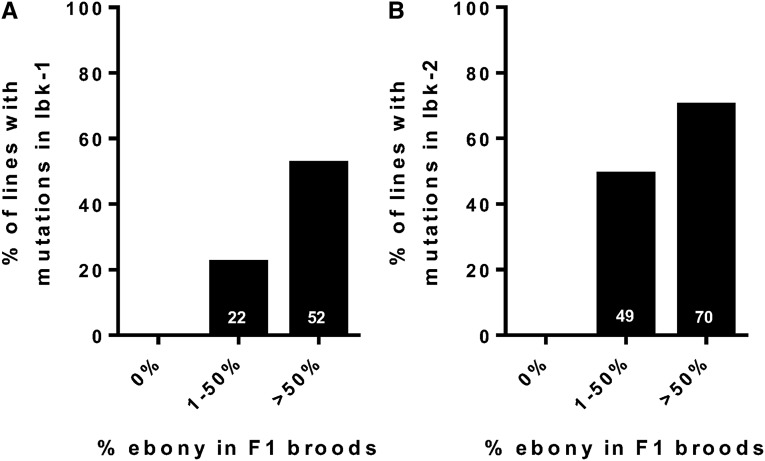
Broods with a high percentage of co-CRISPR marker *e* are highly enriched for mutations in *lbk*. We plot broods from the F1 generation in three groups (0% ebony, 1–50% ebony and >50% ebony) against the percentage of F1-derived balanced with lbk-1 (A) and lbk-2 (B) mutations. We observed a strong correlation between the percentage of *e* in F1 broods and mutations in *lbk* target sites (*P* < 0.05 for lbk-1 and *P* < 0.01 for lbk-2; Pearson correlation: *r*^2^ = 0.21 for lbk-1 mutants, *r*^2^ = 0.32 for lbk-2 mutants). Total numbers of lines sequenced for lbk-1 were 24 (0%), 54 (1–50%), and 42 (>50%), and 24 (0%), 51 (1–50%) and 40 (>50%) for lbk-2.

**Figure 4:**
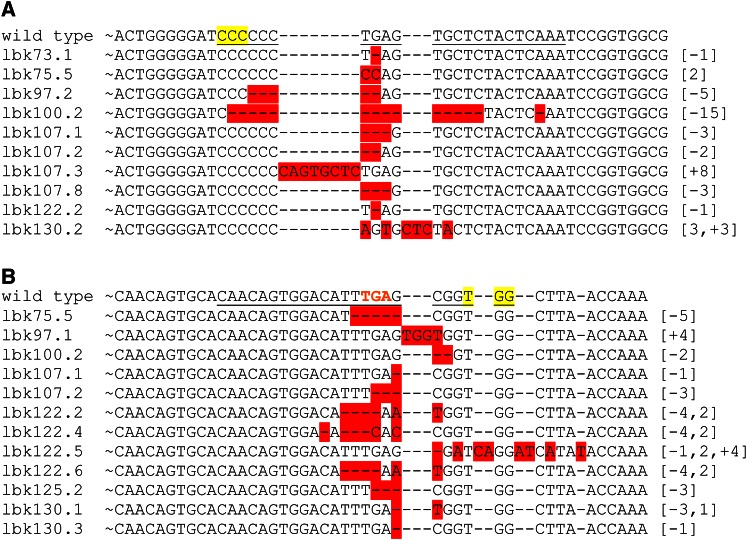
CRISPR-induced mutations in *lbk*. Sequence alignments of wild-type *lbk* in transgenic nos-Cas9 flies and some representative lines of the ebony-enriched jackpot lines containing indels in genomic regions lbk-1 (A) and lbk-2 (B). The PAM sequence is highlighted, with genomic targets underlined. The number of base pairs deleted [−#], inserted [+#] and/or substituted [#] are indicated in brackets. A total of 120 balanced lines were sequenced (235 sequences for the two genomic regions); additional sequence alignments are in Figure S2. The stop codon in lbk-2 in this and subsequent figures is shown in red text.

Approximately 61% of the jackpot lines possessed indels in either lbk-1 (52%) or lbk-2 (70%), and approximately one-third (36%) contained indels in both loci (we found no deletions of the intervening region) (Figure 3, Figure 4, Figure S2, and Table 1B). Significantly, none of the 24 *e+* lines had mutations; all were wild type at the two *lbk* target loci. As expected, broods with a lower number of *e* flies (1–50%) showed an intermediate number of mutations at lbk-1 (22%) and lbk-2 (49%) (Figure 3 and Table 1B). The majority of mutations were small deletions, but we also found substitutions, small insertions, and a few larger deletions and insertions. For both target sites, we observed that ∼67% of the modifications were deletions (median 2 bp, 95% CI, 1.44–11.68 for lbk-1; median 3 bp, 95% CI, 2.46–5.37 for lbk-2) while a mixture of indels (including substitutions) comprised ∼21% of remaining modification (median 6 bp, 95% CI, 4.07–7.06 for lbk-1; median 5.5 bp, 95% CI, 3.14–7.85 for lbk-2) (Figure S2). We found that sibling lines frequently, but not always, contained the same mutations, suggesting that some mosaicism exists in the germlines of injected flies (nine of 11 for lbk-1 and five of 10 lines for lbk-2) (Figure S2 and Table S2). For example, *lbk^73.1^*- *lbk^73.8^* all come from injected parent number 73 but from different, individual *e* F1 males. *lbk^73.1^*, *lbk^73.2^*, *lbk^73.5^*, *lbk^73.7^*, and *lbk^73.8^* have the same 1 bp deletion in lbk-1, but *lbk^73.3^* is wild type in this region. Similarly, the six sibling lines *lbk^130.1– 130.6^* have different mutations from one another in both regions lbk-1 and lbk-2 (Figure S2, A and B).

Seven balanced lines from the collection were homozygous lethal and were rebalanced over *CyO*, *GFP* so that homozygous embryos or larvae could be collected for molecular analysis. We found that all of these lines were larval lethal, and homozygous (non-GFP) L2 larvae were collected to identify potentially lethal mutations of *lbk* targeted by our gRNAs. Sequence analysis of the lbk-1 and lbk-2 genomic regions showed that the homozygous lethal lines were either wild type at these loci or contained the same variety of mutations as the homozygous viable lines we sequenced (Figure S2). For example, sibling lines *lbk^125.1^*, *lbk^125.2^*, and *lbk^125.4^* are all homozygous lethal and have the same 3 bp deletion in the lbk-2 region, but their sibling line *lbk^125.3^* is homozygous viable and has the identical 3 bp deletion (Figure S2B). Therefore, we believe that the lethality is not due to *lbk* mutations but rather to some unknown lethal mutation on chromosome 2 or an off-target effect of CRISPR-Cas9.

### HDR for knock-ins

In order to determine whether the use of *e* as a co-CRISPR marker would be useful in identifying HDR knock-in events, nos-Cas9 transgenic embryos were injected with a mix of gRNA-e, gRNA-lbk2, and a repair template plasmid in order to insert a FLAG-HA tag at the C-terminus of *lbk*. Seventy-two balanced lines were generated from individual *e* F1 males or virgin females from 17 separate broods, and PCR was performed to check for HDR knock-in events in all 72 lines (Table 2A), which would appear as a 225 bp insertion at the end of *lbk* (Figure 1). We began by testing lines from the two best jackpot broods, which had 74 and 81% *e*. As we had done previously, we attempted to establish six lines from each of the two broods, resulting in 11 fertile lines. Two of these 11 lines (18%) had an HDR insertion, as confirmed by sequencing. Analysis of the remaining jackpot lines from broods with >50% *e* revealed another HDR knock-in. Additionally, a fourth HDR insertion line was identified from a low *e* brood (Table 2B). The four lines with HDR insertions derived from broods that had 74, 74, 51, and 19% *e*. Two of the lines with insertions were sibling lines from the same P, from a brood with 74% *e*.

## Discussion

The ease and accuracy of CRISPR-Cas9–based technology is revolutionizing basic research and has great potential in medical science. The reported frequencies of CRISPR-induced changes in *Drosophila* vary greatly, with the rate limiting step being identification of modified genomes, typically involving PCR amplification of target regions or mismatch-detection through endonuclease assays followed by sequencing (Bassett and Liu 2014). In the cases where the editing event is rare or where DNA modifications lead to an unknown change in phenotype, the screening process can be very time-consuming or laborious. The same is true for in frame knock-ins where endogenous expression of the target gene may be below the threshold for easy detection using conventional microscopic methods or standard biochemistry. In some cases, it is useful to use a DsRed knock-in in a mutated gene for identification of some CRISPR events (Gratz *et al.* 2013). In *C. elegans*, three groups have shown that by identifying animals that have a co-CRISPR or coconversion marker mutation, the probability of having a CRISPR-induced change in a gene of interest in the same cell is also high (Arribere *et al.* 2014; Kim *et al.* 2014; Ward 2015). In this work we show that a co-CRISPR technique in *Drosophila* also saves time and labor for identifying CRISPR-induced mutations.

In order to identify a useful co-CRISPR marker in *Drosophila*, we examined the utility of many mutations that are easily scorable. Dominant markers in flies exist, but often result in a lethal phenotype when homozygous. Based on these considerations, we chose a recessive, homozygous viable mutation, *e*, which has an easily scorable phenotype, and is not part of common transgenic constructs. We reasoned that the ideal co-CRISPR mutations needed to be generated at an intermediate frequency within and among the broods. A very high frequency of the marker phenotype would diminish its usefulness in identifying broods with a co-CRISPR event, and a poor frequency of conversion would likely lead to a failure in detecting CRISPR mutations in the gene of interest. During the preparation of our manuscript, a study in *Drosophila* was published using the *white* (*w*) gene as a co-CRISPR marker (Ge *et al.* 2016). The advantages of the *w* gene as a co-CRISPR marker are similar to the *e* gene used in our study. However, the *w* gene is commonly used as a transgenic marker in flies, necessitating the generation of the appropriate fly stocks in order to use *w* as a co-CRISPR marker. Importantly, *w* is a marker commonly used to identify fly stocks with Cas9 insertions (Harrison *et al.* 2014; Port *et al.* 2014). The *e* gene is not used in fly transgenics, eliminating the need to generate specialized stocks for using it as a co-CRISPR marker. Thus, we believe that *e* is a robust co-CRISPR marker for many experiments.

Our data show that using the *e* gene as a co-CRISPR marker significantly reduces the effort required to identify mutations in a gene of interest in *Drosophila*. We show that jackpot broods in the F1 generation are more highly enriched for mutations in the gene of interest (52 and 70%, respectively, for the two target sites used). Mutations generated at target sites included mainly small deletions and also included substitutions, small insertions, and a few larger indels, similar to what has been previously observed (Figure 4 and Figure S2) (Bassett and Liu 2014; Gratz *et al.* 2013; Kondo and Ueda 2013; Waaijers *et al.* 2013; Wang *et al.* 2013). Lines with only insertions or substitutions were rare for both target sites (<6% of all lines tested). Of note, we also observe a 36% instance of both target sites being mutated in jackpot broods (Table 1B), indicating the usefulness of our methodology to target more than one target site at a time. This approach of using two guides in one gene allowed the possibility of recovering deletions between them (Kondo and Ueda 2013). Two possible explanations for why we did not recover these rarer deletion events include the larger distance between the two guides (1.6 *vs.* 4.2 kb) and/or that we only sampled a limited number of flies in each brood for these events.

Tagging a gene of interest (*e.g.*, FLAG-HA, GFP, or TagRFP) can be useful for biochemical and cell biological studies at the endogenous level, but these events can be quite rare because it involves HDR events. We show that the identification of knock-in HDR events is also enriched in jackpot broods: we detected two knock-ins from the first 11 flies tested for integration of a FLAG-HA cassette at the C-terminus of the *lbk* gene (Table 2B). Given the low number of recovered HDR events and the lack of analysis of the zero ebony broods for HDR events, we cannot unambiguously conclude that ebony-containing broods will identify second HDR events. However, in two previous *C. elegans* co-CRISPR studies, there is a strong correlation between the co-CRISPR marker and a second CRISPR event (Arribere *et al.* 2014; Kim *et al.* 2014). Based on the data in our NHEJ CRISPR experiments and the results from similar experiments in *C. elegans*, we suggest focusing on jackpot broods for identification of HDR events in *Drosophila*.

Promoters currently used to drive Cas9 expression in flies include *vasa*, *actin*, and *nos* (Bassett *et al.* 2013; Kondo and Ueda 2013; Port *et al.* 2014). The *vasa* and *actin* promoters are active in the soma as well as in the germline. The advantage of utilizing these promoters to drive Cas9 expression is that co-CRISPR events may be identified in the injected P generation itself by observing mosaicism. The use of these promoters is beneficial only if modifications in the gene of interest are not lethal. Given that many genes lead to somatic lethality in the fly, we chose to use the *nos* promoter to drive expression of Cas9—a promoter that is active mainly in the germline (Port *et al.* 2015). By utilizing this approach, one ensures that potentially lethal mutations in the gene of interest will be recovered (see Figure 2 and *Materials and Methods* for details).

The cellular mechanisms and/or variables during injection leading to the generation of a jackpot brood are not well understood. Nonetheless, co-CRISPR techniques have conclusively shown that jackpot broods are enriched for modification events at the gene of interest. Focusing on these broods, rather than random screening, substantially reduces the need to perform extensive molecular analysis of progeny to identify mutants. In conclusion, selecting for flies mutated in the *e* gene when using co-CRISPR will allow for the rapid screening and identification of CRISPR-induced events within the gene of interest. We anticipate that this technique will be a valuable tool in the modification of genes in *Drosophila*.

## Supplementary Material

Supplemental material is available online at www.g3journal.org/lookup/suppl/doi:10.1534/g3.116.036723/-/DC1.
