# Supplementary material for: Efficient Screening of CRISPR/Cas9-Induced Events in Drosophila Using a Co-CRISPR Strategy
Source: G3 (Bethesda). 2016 Oct 28;7(1):87–93. doi: 10.1534/g3.116.036723 (PMC5217126; doi:10.1534/g3.116.036723)
Supplement: Supplementary file 1 [file 87FigureS1.docx]

**Figure S1: Sequence of HDR dsDNA repair template for *lbk* knock-in.**

5’-CAGACGGACTTGGAGGCCCAGCAGGATCATTTGTCCAGCAAGGACTCGGGCACCGGTTCGGA

TGCGGCTGTGAAACGCACTCTGGATGACTTTGAGGTGGCCATGCCCAGTCATAAGCATCACACCG

ACGACGAGGAGGAGGAGGAGGTTGAGGAGGTGTATGAAGAAAACGAGCCACCATACATAAAGCAT

ACATACGAAGTGAATTATGCTGAACCCGAGCAGCATCTCTTTCTGCAGAACAACAATCACAACTA

CGACGGTGGCGGGATTGGCGTTGCTGGTGGGGTCAACAAAATGGTGCCCAACGCGCTGCTCCGCA

AATGCAGCGCTGGTGCCAGCGGCAGCAACTACAGCTCCATCCAACAGTGCAC**AACAGTGGACATT**

**ATGGACTACAAAGACCATGACGGTGATTATAAAGATCATGACATCGATTACAAGGATGACGATGA**

**CAAGCACCGGTTGAGCTCCGCCACCATGGATCTCCACCGCGGTGGAGGCCGCATCTTTTACCCAT**

**ACGATGTTCCTGACTATGCGGGCTATCCCTATGACGTCCCGGACTATGCAGGATCCTATCCATAT**

**GACGTTCCAGATTACGCTGCTCATGGCGGATGAGCCATCA**CTTAACCAAATGAGCAGGATGATCA

GGATCATATACCAAAAACTGGAGGCTGCACCGTAACCCCAACCGCAGTCCGCCCCCAAAACGAAC

ACAAAGACTGTTTAACTCGTAATTATAATTCGTAGATGGTGGCCCAGACTTTTGGACTGGCCACG

ACCATAGAGCAGGCGGCCTTATTGATGCCTTTGCTATGGAAATAGGACAGCTAGAGAAACATACC

AGTGTCTTCGTAATTTATGAAATTAATCGTTAGAATCGATTATCATTAACTTGTAATGCATAATA

TTTAATTATCGTATATGTATATTTATAATTTTCTTTCGCTTGTGATATTATTTATTATTTTGATT

GCCCGAGTTTTAGACGTAATACATTCA

The repair template consists of a 225 bp 3xFLAG-3X-HA flanked by 387 and 384 bp *lbk* homology arms (Fig. 1). This repair template was synthesized as a gene block (gB) by Integrated DNA Technologies, Inc., PCR amplified, and cloned into TOPO^®^ vector. The gRNA-lbk2 genomic target is underlined. The 3xFLAG-3xHA epitope tag is blue, the stop codon is red, followed by mutations at the PAM site of two GG changed to CA shown in green text.
