# Supplementary material for: Efficient Screening of CRISPR/Cas9-Induced Events in Drosophila Using a Co-CRISPR Strategy
Source: G3 (Bethesda). 2016 Oct 28;7(1):87–93. doi: 10.1534/g3.116.036723 (PMC5217126; doi:10.1534/g3.116.036723)
Supplement: Supplementary file 2 [file 87FigureS2.docx]

**Figure S2: CRISPR-induced mutations in *lbk.***

**A**

Wild type ~ACTGGGGGATCCCCCC--------TGAG---TG-----CTCTACTCAAATCCGGTGGCG

lbk70.1 ~ACTGGGGGATCCCCCC--------TGAG---TG-----CTCTACTCAAATCCGGTGGCG

lbk70.2 ~ACTGGGGGATCCCCCC--------TGAG---TG-----CTCTACTCAAATCCGGTGGCG

lbk70.3 ~ACTGGGGGATCCCCCC--------TGAG---TG-----CTCTACTCAAATCCGGTGGCG

lbk70.4 ~ACTGGGGGATCCCCCC--------TGAG---TG-----CTCTACTCAAATCCGGTGGCG

lbk70.5 ~ACTGGGGGATCCCCCC----------AG---TA-----CTCTACTCAAATCCGGTGGCG [1]

lbk73.1 ~ACTGGGGGATCCCCCC--------T-AG---TG-----CTCTACTCAAATCCGGTGGCG [-1]

lbk73.2 ~ACTGGGGGATCCCCCC--------T-AG---TG-----CTCTACTCAAATCCGGTGGCG [-1]

lbk73.3 ~ACTGGGGGATCCCCCC--------TGAG---TG-----CTCTACTCAAATCCGGTGGCG

lbk73.5 ~ACTGGGGGATCCCCCC--------T-AG---TG-----CTCTACTCAAATCCGGTGGCG [-1]

lbk73.7 ~ACTGGGGGATCCCCCC--------T-AG---TG-----CTCTACTCAAATCCGGTGGCG [-1]

lbk73.8 ~ACTGGGGGATCCCCCC--------T-AG---TG-----CTCTACTCAAATCCGGTGGCG [-1]

lbk75.5 ~ACTGGGGGATCCCCCC--------CCAG---TG-----CTCTACTCAAATCCGGTGGCG [2]

lbk96.1 ~ACTGGGGGATCCCCCC--------TGAG---TG-----CTCTACTCAAATCCGGTGGCG

lbk96.4 ~ACTGGGGGATCCCCCC--------TGAG---TG-----CTCTACTCAAATCCGGTGGCG

lbk97.1 ~ACTGGGGGATCCCCCC--------TGAG---TG-----CTCTACTCAAATCCGGTGGCG

lbk97.2 ~ACTGGGGGATCCC-------------AG---TG-----CTCTACTCAAATCCGGTGGCG [-5]

lbk100.2 ~ACTGGGGGATC------------------------------TACTC-AATCCGGTGGCG [-15]

lbk100.3 ~ACTGGGGGATC------------------------------TACTC-AATCCGGTGGCG [-15]

lbk107.1 ~ACTGGGGGATCCCCCC-----------G---TG-----CTCTACTCAAATCCGGTGGCG [-3]

lbk107.2 ~ACTGGGGGATCCCCCC----------AG---TG-----CTCTACTCAAATCCGGTGGCG [-2]

lbk107.3 ~ACTGGGGGATCCCCCCCAGTGCTCTGAG---TG-----CTCTACTCAAATCCGGTGGCG [+8]

lbk107.4 ~ACTGGGGGATCCCCCC--------TGAG---TG-----CTCTACTCAAATCCGGTGGCG

lbk107.8 ~ACTGGGGGATCCCCCC-----------G---TG-----CTCTACTCAAATCCGGTGGCG [-3]

lbk107.9 ~ACTGGGGGATCCCCCC--------TGAG---TG-----CTCTACTCAAATCCGGTGGCG

lbk122.1 ~ACTGGGGGATCCCCCC--------TGAG---TG-----CTCTACTCAAATCCGGTGGCG

lbk122.2 ~ACTGGGGGATCCCCCC--------T-AG---TG-----CTCTACTCAAATCCGGTGGCG [-1]

lbk122.3 ~ACTGGGGGATCCCCCC--------TGAG---TG-----CTCTACTCAAATCCGGTGGCG

lbk122.4 ~ACTGGGGGATCCCCCC--------TGAG---TG-----CTCTACTCAAATCCGGTGGCG

lbk122.5 ~ACTGGGGGATCCCCCC--------T-AG---TG-----CTCTACTCAAATCCGGTGGCG [-1]

lbk122.6 ~ACTGGGGGATCCCCCC--------TGAG---TG-----CTCTACTCAAATCCGGTGGCG

lbk125.1* ~ACTGGGGGATCCCCCC--------TGAG---TG-----CTCTACTCAAATCCGGTGGCG

lbk125.2* ~ACTGGGGGATCCCCCC--------TGAG---TG-----CTCTACTCAAATCCGGTGGCG

lbk125.3 ~ACTGGGGGATCCCCCC--------TGAG---TG-----CTCTACTCAAATCCGGTGGCG

lbk125.4* ~ACTGGGGGATCCCCCC--------TGAG---TG-----CTCTACTCAAATCCGGTGGCG

lbk125.6 ~ACTGGGGGATCCCCCC--------TGAG---TG-----CTCTACTCAAATCCGGTGGCG

lbk130.1 ~ACTGGGGGATCCCCCC----------AG---TGCTCTACTCTACTCAAATCCGGTGGCG [-2,+5]

lbk130.2 ~ACTGGGGGATCCCCCC--------AGTGCTCTA-----CTCTACTCAAATCCGGTGGCG [3,+3]

lbk130.3 ~ACTGGGGGATCCCCCC--------AGTGCTCTA-----CTCTACTCAAATCCGGTGGCG [3,+3]

lbk130.4 ~ACTGGGGGATCCCCCC--------AGTGCTCTA-----CTCTACTCAAATCCGGTGGCG [3,+3]

lbk130.5 ~ACTGGGGGATCCCCCC--------AGTGCTCTA-----CTCTACTCAAATCCGGTGGCG [3,+3]

lbk130.6 ~ACTGGGGGATCCCCCC--------AGTGCTCTA-----CTCTACTCAAATCCGGTGGCG [3,+3]

lbk152.2 ~ACTGGGGGATCCCCCC--------TGAG---TG-----CTCTACTCAAATCCGGTGGCG

**B**

Wild type ~CAACAGTGCACAACAGTGGACATT**TGA-**G---CGGT--GG--CTTA-ACCAAA

lbk70.1 ~CAACAGTGCACAACAGTGGACATTTGATG—-ATGGT--GG--CTTA-ACCAAA [+2,1]

lbk70.2 ~CAACAGTGCACAACAGTGGACATTTGATG—-ATGGT--GG--CTTA-ACCAAA [+2,1]

lbk70.4 ~CAACAGTGCACAACAGTGGACATTTGATG—-ATGGT--GG--CTTA-ACCAAA [+2,1]

lbk70.5 ~CAACAGTGCACAACAGTGGACATTTGA--------T--GG--CTTA-ACCAAA [-4]

lbk73.1 ~CAACAGTGCACAACAGTGGACATTTGA-G---CGGT--GG--CTTA-ACCAAA

lbk73.2 ~CAACAGTGCACAACAGTGGACATTTGA-G---CGGT--GG--CTTA-ACCAAA

lbk73.3 ~CAACAGTGCACAACAGTGGACATTTGA-G---CGGT--GG--CTTA-ACCAAA

lbk73.5 ~CAACAGTGCACAACAGTGGACATTTGA-G---CGGT--GG--CTTA-ACCAAA

lbk73.7 ~CAACAGTGCACAACAGTGGACATTTGA-G---CGGT--GG--CTTA-ACCAAA

lbk73.8 ~CAACAGTGCACAACAGTGGACATTTGA-G---CGGT--GG--CTTA-ACCAAA

lbk75.5 ~CAACAGTGCACAACAGTGGACAT---------CGGT--GG--CTTA-ACCAAA [-5]

lbk96.1 ~CAACAGTGCACAACAGTGGACATTTGA-G---CGGT--GG--CTTA-ACCAAA

lbk96.4 ~CAACAGTGCACAACAGTGGACATTTGA-G---CGGT--GG--CTTA-ACCAAA

lbk97.1 ~CAACAGTGCACAACAGTGGACATTTGA-GTGGTGGT--GG--CTTA-ACCAAA [+4]

lbk97.2 ~CAACAGTGCACAACAGTGGACATTTGA-GTGGTGGT--GG--CTTA-ACCAAA [+4]

lbk100.2 ~CAACAGTGCACAACAGTGGACATTTGA-G-----GT--GG--CTTA-ACCAAA [-2,1]

lbk100.3 ~CAACAGTGCACAACAGTGGACATTTGA-G-----GT--GG--CTTA-CCCAAA

lbk107.1 ~CAACAGTGCACAACAGTGGACATTTGA-----CGGT--GG--CTTA-ACCAAA [-1]

lbk107.2 ~CAACAGTGCACAACAGTGGACATTT-------CGGT--GG--CTTA-ACCAAA [-3]

lbk107.3 ~CAACAGTGCACAACAGTGGACATTT-------CGGT--GG--CTTA-ACCAAA [-3]

lbk107.4 ~CAACAGTGCACAACAGTGGACATTT-------CGGT--GG--CTTA-ACCAAA [-3]

lbk107.8 ~CAACAGTGCACAACAGTGGACATTTGA-----CGGT--GG--CTTA-ACCAAA [-1]

lbk107.9 ~CAACAGTGCACAACAGTGGACATTT-------CGGT--GG--CTTA-ACCAAA [-3]

lbk122.1 ~CAACAGTGCACAACAGTGGACATTTGA-G---CGGT--GG--CTTA-CCCAAA

lbk122.2 ~CAACAGTGCACAACAGTGGACA----A-A---TGGT--GG--CTTA-ACCAAA [-4,2]

lbk122.3 ~CAACAGTGCACAACAGTGGACA----A-A---TGGT--GG--CTTA-ACCAAA [-4,2]

lbk122.4 ~CAACAGTGCACAACAGTGGA-A---CA-C---CGGT--GG--CTTA-ACCAAA [-4,2]

lbk122.5 ~CAACAGTGCACAACAGTGGACATTTGA-G----GATCAGGATCATATACCAAA [-1,+5,1]

lbk122.6 ~CAACAGTGCACAACAGTGGACA----A-A---TGGT--GG--CTTA-ACCAAA [-4,2]

lbk125.1* ~CAACAGTGCACAACAGTGGACATTT-------CGGT--GG--CTTA-ACCAAA [-3]

lbk125.2* ~CAACAGTGCACAACAGTGGACATTT-------CGGT--GG--CTTA-ACCAAA [-3]

lbk125.3 ~CAACAGTGCACAACAGTGGACATTT-------CGGT--GG--CTTA-ACCAAA [-3]

lbk125.4* ~CAACAGTGCACAACAGTGGACATTT-------CGGT--GG--CTTA-ACCAAA [-3]

lbk125.6 ~CAACAGTGCACAACAGTGGACATTT-------CGGT--GG--CTTA-ACCAAA [-3]

lbk130.1 ~CAACAGTGCACAACAGTGGACATTTGA-----TGGT--GG--CTTA-ACCAAA [-1,1]

lbk130.2 ~CAACAGTGCACAACAGTGGACATTTGA-G---CGGT--GG--CTTA-ACCAAA

lbk130.3 ~CAACAGTGCACAACAGTGGACATTTGA-----CGGT--GG--CTTA-ACCAAA [-1]

lbk130.4 ~CAACAGTGCACAACAGTGGACATTTGA-G---CGGT--GG--CTTA-ACCAAA

lbk130.5 ~CAACAGTGCACAACAGTGGACATTTGA-----CGGT--GG--CTTA-ACCAAA [-1]

lbk130.6 ~CAACAGTGCACAACAGTGGACATTTGA-G---CGGT--GG--CTTA-ACCAAA

**C**

wild type ~ACTGGGGGATCCCCCC-TGAGTGCTCTACTCAAATCCGGTGGCG

lbk63.1 ~ACTGGGGGATCCCCCC--GAGTGCTCTACTCAAATCCGGTGGCG [-1]

lbk63.2 ~ACTGGGGGATCCCCCC-TGAGTGCTCTACTCAAATCCGGTGGCG

lbk63.3 ~ACTGGGGGATCCCCC----AGTGCTCTACTCAAATCCGGTGGCG [-3]

lbk63.4 ~ACTGGGGGATCCCCCC-TGAGTGCTCTACTCAAATCCGGTGGCG

lbk63.5 ~ACTGGGGGATCCCCCC-TGAGTGCTCTACTCAAATCCGGTGGCG

lbk63.6 ~---------T-------TGAGTGCTCTACTCAAATCCGGTGGCG [-42]

lbk63.7 ~---------T-------TGAGTGCTCTACTCAAATCCGGTGGCG [-42]

lbk81.3 ~ACTGGGGGATCCCCCC-TGAGTGCTCTACTCAAATCCGGTGGCG

lbk82.1* ~ACTGGGGGATCCCCCC-TGAGTGCTCTACTCAAATCCGGTGGCG

lbk82.3* ~ACTGGGGGATCCCCCC-TGAGTGCTCTACTCAAATCCGGTGGCG

lbk88.1 ~ACTGGGGGATCCCCCC-TGAGTGCTCTACTCAAATCCGGTGGCG

lbk89.1 ~ACTGGGGGATCCCCCC-TGAGTGCTCTACTCAAATCCGGTGGCG

lbk91.1 ~ACTGGGGGATCCCCCC-TGAGTGCTCTACTCAAATCCGGTGGCG

lbk91.2 ~ACTGGGGGATCCCCCC---AGTGCTCTACTCAAATCCGGTGGCG [-2]

lbk91.3 ~ACTGGGGGATCCCCCC-TGAGTGCTCTACTCAAATCCGGTGGCG

lbk91.4 ~ACTGGGGGATCCCCCC-TGAGTGCTCTACTCAAATCCGGTGGCG

lbk119.1 ~ACTGGGGGATCCCCCC-TGAGTGCTCTACTCAAATCCGGTGGCG

lbk119.4 ~ACTGGGGGATCCCCCC-TGAGTGCTCTACTCAAATCCGGTGGCG

lbk119.5 ~ACTGGGGGATCCCCCC-TGAGTGCTCTACTCAAATCCGGTGGCG

lbk128.1 ~ACTGGGGGATCCCCCC---AGTGCTCTACTCAAATCCGGTGGCG [-2]

lbk128.2 ~ACTGGGGGATCCCCCC-TGAGTGCTCTACTCAAATCCGGTGGCG

lbk128.3 ~ACTGGGGGATCCCCCC-TGAGTGCTCTACTCAAATCCGGTGGCG

lbk129.2 ~ACTGGGGGATCCCCCC--CAGTGCTCTACTCAAATCCGGTGGCG [-1,1]

lbk129.3 ~ACTGGGGGATCCCCCC-TGAGTGCTCTACTCAAATCCGGTGGCG

lbk129.4 ~ACTGGGGGATCCCCCC-TGAGTGCTCTACTCAAATCCGGTGGCG

lbk139.1 ~ACTGGGGGATCCCCCC---AGTGCTCTACTCAAATCCGGTGGCG [-2]

lbk139.2 ~ACTGGGGGATCCCCCC-TGAGTGCTCTACTCAAATCCGGTGGCG

lbk139.3 ~ACTGGGGGATCCCCCC-TGAGTGCTCTACTCAAATCCGGTGGCG

lbk139.4 ~ACTGGGGGATCCCCC----AGTGCTCTACTCAAATCCGGTGGCG [-3]

lbk139.5 ~ACTGGGGGATCCCCCC-TGAGTGCTCTACTCAAATCCGGTGGCG

lbk139.6 ~ACTGGGGGATCCCCCC-TGAGTGCTCTACTCAAATCCGGTGGCG

lbk139.7 ~ACTGGGGGATCCCCCC-TGAGTGCTCTACTCAAATCCGGTGGCG

lbk144.1 ~ACTGGGGGATCCCCCC-TGAGTGCTCTACTCAAATCCGGTGGCG

lbk144.2 ~ACTGGGGGATCCCCCC-TGAGTGCTCTACTCAAATCCGGTGGCG

lbk144.4 ~ACTGGGGGATCCCCCC-TGAGTGCTCTACTCAAATCCGGTGGCG

lbk144.5 ~ACTGGGGGATCCCCCC-TGAGTGCTCTACTCAAATCCGGTGGCG

lbk144.6 ~ACTGGGGGATCCCCCC-TGAGTGCTCTACTCAAATCCGGTGGCG

lbk144.9 ~ACTGGGGGATCCCCCC-TGAGTGCTCTACTCAAATCCGGTGGCG

lbk144.10 ~ACTGGGGGATCCCCCC-TGAGTGCTCTACTCAAATCCGGTGGCG

lbk146.1 ~ACTGGGGGATCCCCCC-TGAGTGCTCTACTCAAATCCGGTGGCG

lbk146.2 ~ACTGGGGGATCCCCCC-TGAGTGCTCTACTCAAATCCGGTGGCG

lbk146.3 ~ACTGGGGGATCCCCCC-TGAGTGCTCTACTCAAATCCGGTGGCG

lbk146.4 ~ACTGGGGGATCCCCCC-TGAGTGCTCTACTCAAATCCGGTGGCG

lbk146.5 ~ACTGGGGGATCCCCCC-TGAGTGCTCTACTCAAATCCGGTGGCG

lbk147.1* ~ACTGGGGGATCCCCCC-TGAGTGCTCTACTCAAATCCGGTGGCG

lbk148.2 ~ACTGGGGGATCCCCCC-TGAGTGCTCTACTCAAATCCGGTGGCG

lbk149.1 ~ACTGGGGGATCCCCCCTTGAGTGCTCTACTCAAATCCGGTGGCG [+1]

lbk149.2 ~ACTGGGGGATCCCCCC-TGAGTGCTCTACTCAAATCCGGTGGCG

lbk149.3 ~ACTGGGGGATCCCCCC-TGAGTGCTCTACTCAAATCCGGTGGCG

lbk150.1 ~ACTGGGGGATCCCCCC---AGTGCTCTACTCAAATCCGGTGGCG [-2]

lbk150.2 ~ACTGGGGGATCCCCCC-TGAGTGCTCTACTCAAATCCGGTGGCG

lbk150.4 ~ACTGGGGGATCCCCCC---AGTGCTCTACTCAAATCCGGTGGCG [-2]

lbk151.1 ~ACTGGGGGATCCCCCC-TGAGTGCTCTACTCAAATCCGGTGGCG

lbk151.2 ~ACTGGGGGATCCCCCC-TGAGTGCTCTACTCAAATCCGGTGGCG

**D**

wild type ~AACAGTGCACAACAGTGGA--CA--TT---TG----A-G-----CGGTGGCTTAACCAAA

lbk63.1 ~AACAGTGCACAACAGTGGA--CA--TT---TG----A-G-----TGGTGGCTTAACCAAA [1]

lbk63.2 ~AACAGTGCACAACAGTGGA--CA--TT---TG----A-G-----CGGTGGCTTAACCAAA

lbk63.3 ~AACAGTGCACAACAGTGGA--CA--TT---TG----A-G-----CGGTGGCTTAACCAAA

lbk63.4 ~AACAGTGCACAACAGTGGA--CA--TT---TG----A-G-----CGGTGGCTTAACCAAA

lbk63.6 ~AACAGTGCACAACAGTGGA--CA--TT-----------------CGGTGGCTTAACCAAA [-4]

lbk63.7 ~AACAGTGCACAACAGTGGA--CA--TT-----------------CGGTGGCTTAACCAAA [-4]

lbk82.1* ~AACAGTGCACAACAGTGGA--CA--TT---TG----A-G-----CGGTGGCTTAACCAAA

lbk82.3* ~AACAGTGCACAACAGTGGA--CA--TT---TG----A-G-----CGGTGGCTTAACCAAA

lbk88.1 ~AACAGTGCACAACAGTGGA--CA--TT---TG----A-G------GGTGGCTTAACCAAA [-1]

lbk89.1 ~AACAGTGCACAACAGTGGA--CA--TT---TG----A-G-----CGGTGGCTTAACCAAA

lbk91.1 ~AACAGTGCACAACAGTGGA--CA--TT---TG----A-------CGGTGGCTTAACCAAA [-1]

lbk91.2 ~AACAGTGCACAACAGTGGA--CA--TT---TG-----------------GCTTAACCAAA [-7]

lbk91.3 ~AACAGTGCACAACAGTGGA--CA--TT---TG----A-------CGGTGGCTTAACCAAA [-1]

lbk91.4 ~AACAGTGCACAACAGTGGA--CA--TT---TG----A-------CGGTGGCTTAACCAAA [-1]

lbk119.1 ~AACAGTGCACAACAGTGGA--CA-------------A-------CGGTGGCTTAACCAAA [-5]

lbk119.4 ~AACAGTGCACAACAGTGGA--CA-------------A-------CGGTGGCTTAACCAAA [-5]

lbk119.5 ~AACAGTGCACAACAGTGGA--CA-------------A-------CGGTGGCTTAACCAAA [-5]

lbk128.1 ~AACAGTGCACAACAGTGGA--CA--TT---TG----A-G-----CGGTGGCTTAACCAAA

lbk128.2 ~AACAGTGCACAACAGTGGA--CA--TT--------------------TGGCTTAACCAAA [-7]

lbk128.3 ~AACAGTGC-------------------------------------------TTAACCAAA [-26]

lbk129.2 ~AACAGTGCACAACAGTGGA--CA--TT---TG----A-G-----CGGTGGCTTAACCAAA

lbk129.3 ~AACAGTGCACAACAGTGGA--CA--TT---TG----A-G-----CGGTGGCTTAACCAAA

lbk129.4 ~AACAGTGCACAACAGTGGA--CA--TT---TG----A-G-----CGGTGGCTTAACCAAA

lbk139.1 ~AACAGTGCACAACAGTGGA--CA--TT---TG----A-G-----TGGTGGCTTAACCAAA [1]

lbk139.2 ~AACAGTGCACAACAGTGGA--CA--TT---TG----A-G-----TGGTGGCTTAACCAAA [1]

lbk139.3 ~AACAGTGCACAACAGTGGA--CA--TT---TG----A-------CGGTGGCTTAACCAAA [-1]

lbk139.4 ~AACAGTGCACAACAGTGGA--CA--TT---TG----A-G-----CGGTGGCTTAACCAAA

lbk139.5 ~AACAGTGCACAACAGTGGA--CA--TT---TG----A-G-----CGGTGGCTTAACCAAA

lbk139.6 ~AACAGTGCACAACAGTGGA--CA--TT---TG----A-------CGGTGGCTTAACCAAA [-1]

lbk139.7 ~AACAGTGCACAACAGTGGA--CA--TT---TG----A-G-----CGGTGGCTTAACCAAA

lbk144.1 ~AACAGTGCACAACAGTGGA--CA--TT---TG----A-G-----CGGTGGCTTAACCAAA

lbk144.4 ~AACAGTGCACAACAGTGGA--CA--TT---TG----A-G-----CGGTGGCTTAACCAAA

lbk144.5 ~AACAGTGCACAACAGTGGA--CA--TT----G----T-C-----CACTGGCTTAACCAAA [-1,4]

lbk144.6 ~AACAGTGCACAACAGTGGA--CA--TT---TG----A-G-----CGGTGGCTTAACCAAA

lbk144.9 ~AACCGTGCACAACAGTGGA--CA--TT---TG----A---------GTGGCTTAACCAAA [-3]

lbk144.10 ~AACAGTGCACAACAGTGGA--CA--TT---TG----A-G-----CGGTGGCTTAACCAAA

lbk146.1 ~AACAGTGCACAACAGTGGA--CA--TT---TG----A-G-----CGGTGGCTTAACCAAA

lbk146.2 ~AACAGTGCACAACAGTGGA--CA--TT---TG----A-G-----CGGTGGCTTAACCAAA

lbk146.3 ~AACAGTGCACAACAGTGGA--CA--TT---TG----A-G-----CGGTGGCTTAACCAAA

lbk146.4 ~AACAGTGCACAACAGTGGA--CA--TT---TG----A-G-----CGGTGGCTTAACCAAA

lbk146.5 ~AACAGTGCACAACAGTGGA--CA--TT---TG----A-G-----CGGTGGCTTAACCAAA

lbk147.1* ~AACAGTGCACAACAGTGGA--CA--TT---TG----A-G-----CGGTGGCTTAACCAAA

lbk148.2 ~AACAGTGCACAACAGTGGA--CA--TT---TG----A-G-----CGGTGGCTTAACCAAA

lbk149.1 ~AACAGTGCACAACAGTGCA--GTGGTTAAGTGGTTAA-GCCAC-CGGTGGCTTAACCAAA [3,+13]

lbk149.2 ~AACAGTGCACAACAGTGGA--CA--TT---TG----A-G-----CGGTGGCTTAACCAAA

lbk149.3 ~AACAGTGCACAACAGTGGA--CA--TT------------------GGTGGCTTAACCAAA [-5]

lbk150.1 ~AACAGTGCACAACAGTGGA--CA--TT---------------------GGCTTAACCAAA [-7]

lbk150.2 ~AACAGTGCACAACAGTGGA--CA--TT---------------------GGCTTAACCAAA [-7]

lbk150.4 ~AACAGTGCACAACAGTGGA--CA--TT---------------------GGCTTAACCAAA [-7]

lbk151.1 ~AACAGTGCACAACAGTGGA--CA--TT---TG----A-G-----CGGTGGCTTAACCAAA

lbk151.2 ~AACAGTGCACAACAGTGGA--CA--TT---TG----A-------CGGTGGCTTAACCAAA [-1]

**E**

wild type ~ACTGGGGGATCCCCCCTGAGTGCTCTACTCAAATCCGGTGGCG

lbk85.1* ~ACTGGGGGATCCCCCCTGAGTGCTCTACTCAAATCCGGTGGCG

lbk85.2 ~ACTGGGGGATCCCCCCTGAGTGCTCTACTCAAATCCGGTGGCG

lbk85.3 ~ACTGGGGGATCCCCCCTGAGTGCTCTACTCAAATCCGGTGGCG

lbk85.4 ~ACTGGGGGATCCCCCCTGAGTGCTCTACTCAAATCCGGTGGCG

lbk85.5 ~ACTGGGGGATCCCCCCTGAGTGCTCTACTCAAATCCGGTGGCG

lbk85.6 ~ACTGGGGGATCCCCCCTGAGTGCTCTACTCAAATCCGGTGGCG

lbk92.1 ~ACTGGGGGATCCCCCCTGAGTGCTCTACTCAAATCCGGTGGCG

lbk92.2 ~ACTGGGGGATCCCCCCTGAGTGCTCTACTCAAATCCGGTGGCG

lbk92.3 ~ACTGGGGGATCCCCCCTGAGTGCTCTACTCAAATCCGGTGGCG

lbk92.4 ~ACTGGGGGATCCCCCCTGAGTGCTCTACTCAAATCCGGTGGCG

lbk92.5 ~ACTGGGGGATCCCCCCTGAGTGCTCTACTCAAATCCGGTGGCG

lbk92.6 ~ACTGGGGGATCCCCCCTGAGTGCTCTACTCAAATCCGGTGGCG

lbk103.1 ~ACTGGGGGATCCCCCCTGAGTGCTCTACTCAAATCCGGTGGCG

lbk103.2 ~ACTGGGGGATCCCCCCTGAGTGCTCTACTCAAATCCGGTGGCG

lbk103.3 ~ACTGGGGGATCCCCCCTGAGTGCTCTACTCAAATCCGGTGGCG

lbk103.4 ~ACTGGGGGATCCCCCCTGAGTGCTCTACTCAAATCCGGTGGCG

lbk103.5 ~ACTGGGGGATCCCCCCTGAGTGCTCTACTCAAATCCGGTGGCG

lbk103.6 ~ACTGGGGGATCCCCCCTGAGTGCTCTACTCAAATCCGGTGGCG

lbk131.1 ~ACTGGGGGATCCCCCCTGAGTGCTCTACTCAAATCCGGTGGCG

lbk131.2 ~ACTGGGGGATCCCCCCTGAGTGCTCTACTCAAATCCGGTGGCG

lbk131.3 ~ACTGGGGGATCCCCCCTGAGTGCTCTACTCAAATCCGGTGGCG

lbk131.4 ~ACTGGGGGATCCCCCCTGAGTGCTCTACTCAAATCCGGTGGCG

lbk131.5 ~ACTGGGGGATCCCCCCTGAGTGCTCTACTCAAATCCGGTGGCG

lbk131.6 ~ACTGGGGGATCCCCCCTGAGTGCTCTACTCAAATCCGGTGGCG

**F**

wild type ~CAACAGTGCACAACAGTGGACATT**TGA**GCGGTGGCTTAACCAAA

lbk85.1* ~CAACAGTGCACAACAGTGGACATTTGAGCGGTGGCTTAACCAAA

lbk85.2 ~CAACAGTGCACAACAGTGGACATTTGAGCGGTGGCTTAACCAAA

lbk85.3 ~CAACAGTGCACAACAGTGGACATTTGAGCGGTGGCTTAACCAAA

lbk85.4 ~CAACAGTGCACAACAGTGGACATTTGAGCGGTGGCTTAACCAAA

lbk85.5 ~CAACAGTGCACAACAGTGGACATTTGAGCGGTGGCTTAACCAAA

lbk85.6 ~CAACAGTGCACAACAGTGGACATTTGAGCGGTGGCTTAACCAAA

lbk92.1 ~CAACAGTGCACAACAGTGGACATTTGAGCGGTGGCTTAACCAAA

lbk92.2 ~CAACAGTGCACAACAGTGGACATTTGAGCGGTGGCTTAACCAAA

lbk92.3 ~CAACAGTGCACAACAGTGGACATTTGAGCGGTGGCTTAACCAAA

lbk92.4 ~CAACAGTGCACAACAGTGGACATTTGAGCGGTGGCTTAACCAAA

lbk92.5 ~CAACAGTGCACAACAGTGGACATTTGAGCGGTGGCTTAACCAAA

lbk92.6 ~CAACAGTGCACAACAGTGGACATTTGAGCGGTGGCTTAACCAAA

lbk103.1 ~CAACAGTGCACAACAGTGGACATTTGAGCGGTGGCTTAACCAAA

lbk103.2 ~CAACAGTGCACAACAGTGGACATTTGAGCGGTGGCTTAACCAAA

lbk103.3 ~CAACAGTGCACAACAGTGGACATTTGAGCGGTGGCTTAACCAAA

lbk103.4 ~CAACAGTGCACAACAGTGGACATTTGAGCGGTGGCTTAACCAAA

lbk103.5 ~CAACAGTGCACAACAGTGGACATTTGAGCGGTGGCTTAACCAAA

lbk103.6 ~CAACAGTGCACAACAGTGGACATTTGAGCGGTGGCTTAACCAAA

lbk131.1 ~CAACAGTGCACAACAGTGGACATTTGAGCGGTGGCTTAACCAAA

lbk131.2 ~CAACAGTGCACAACAGTGGACATTTGAGCGGTGGCTTAACCAAA

lbk131.3 ~CAACAGTGCACAACAGTGGACATTTGAGCGGTGGCTTAACCAAA

lbk131.4 ~CAACAGTGCACAACAGTGGACATTTGAGCGGTGGCTTAACCAAA

lbk131.5 ~CAACAGTGCACAACAGTGGACATTTGAGCGGTGGCTTAACCAAA

lbk131.6 ~CAACAGTGCACAACAGTGGACATTTGAGCGGTGGCTTAACCAAA

**Figure S2: CRISPR-induced mutations in *lbk.*** Sequence alignments of genomic regions lbk-1 and lbk-2 in wild type (transgenic nos-Cas9) and all balanced lines tested. The PAM sequence is highlighted. Number of base pairs deleted [-#], inserted [+#] and substituted [#] are indicated in brackets and highlighted in red. No numbers in brackets indicate wild-type sequence. Wild-type genomic regions lbk-1 and lbk-2 are underlined. The stop codon is shown in red text. **(A)** lbk-1 and **(B)** lbk-2 sequence alignments in jackpot lines, or broods with >50% *e*. **(C)** lbk-1 and **(D)** lbk-2 sequence alignments in broods with 1-50% *e*. **(E)** lbk-1 and **(F)** lbk-2 sequence alignments in broods with no *e*. Homozygous lethal lines are indicated by *.
