# Supplementary material for: Efficient Screening of CRISPR/Cas9-Induced Events in Drosophila Using a Co-CRISPR Strategy
Source: G3 (Bethesda). 2016 Oct 28;7(1):87–93. doi: 10.1534/g3.116.036723 (PMC5217126; doi:10.1534/g3.116.036723)
Supplement: Supplementary file 3 [file 87TableS1.docx]

**Table S1: Sequences of genomic target sites, oligonucleotides, primers and repair**

**Genomic target sites, oligonucleotides, primers.**

| **gRNA genomic target sites** |  |
| --- | --- |
| **gRNA-e target** | GCCACAATTGTCGATCGTCAAGG |
| **gRNA-lbk1 target** | TTTGAGTAGAGCACTCAGGGGGG |
| **gRNA-lbk2 target** | AACAGTGGACATT**TGA**GCGGTGG |
| **Oligos used for cloning into pCFD3 vector** |  |
| **e Fwd** | GTCGCCACAATTGTCGATCGTCA |
| **e Rev** | AAACTGACGATCGACAATTGTGGC |
| **lbk-1 Fwd** | GTCGTTTGAGTAGAGCACTCAGGG |
| **lbk-1-Rev** | AAACCCCTGAGTGCTCTACTCAAAC |
| **lbk-2 Fwd** | GTCGAACAGTGGACATT**TGA**GCGG |
| **lbk-2 Rev** | AAACCCGCTCAAATGTCCACTGTTC |
| **Primers used for PCR and sequencing** |  |
| **lbk-1 Fwd 3** | GGCGGCGCAACAACAATAAC |
| **lbk-1 Rev 19** | CACATCCGCATCCGAGTAATCC |
| **lbk-2 Fwd 6** | AGGAGGAGGTTGAGGAGGTG |
| **lbk-2 Rev 8** | AAGTCTGGGCCACCATCTACG |
| **lbk-2 Rev 17** | GTGTTTCCACGCACGAAACTG |
| **Primers used to amplify HDR repair template** |  |
| **lbk gBlock-Fwd** | CAGACGGACTTGGAGGCCCAG |
| **lbk gBlock-Rev** | TGAATGTATTACGTCTAAAAC |

For gRNA genomic target sites, underline indicates PAM site. For oligos used for cloning into the pCFD3 vector, a double underline indicates addition of a Bbs1 site and a bolded TGA is the termination codon of *lbk*. In some cases, depending on genomic sequence, a “C” is added to reverse oligos in order to create the BbsI site. Oligos do not contain a PAM site. *e* sequences and cloning protocols for pCFD3 are from (Port *et al.* 2014).
