# Supplementary material for: Efficient Screening of CRISPR/Cas9-Induced Events in Drosophila Using a Co-CRISPR Strategy
Source: G3 (Bethesda). 2016 Oct 28;7(1):87–93. doi: 10.1534/g3.116.036723 (PMC5217126; doi:10.1534/g3.116.036723)
Supplement: Supplementary file 4 [file 87TableS2.docx]

**Table S2: Mutation rates in F1 lines**.

**A**

|  | **Ebony enriched jackpot line number** | **% *e*** | **lbk-1** | **lbk-2** |
| --- | --- | --- | --- | --- |
| 1 | 73.1 | 100 | Y | N |
| 2 | 73.2 | 100 | Y | N |
| 3 | 73.3 | 100 | N | N |
| 4 | 73.5 | 100 | Y | N |
| 5 | 73.7 | 100 | Y | N |
| 6 | 73.8 | 100 | Y | N |
| 7 | 75.5 | 100 | Y | Y |
| 8 | 100.2 | 100 | Y | Y |
| 9 | 100.3 | 100 | Y | Y |
| 10 | 125.1* | 91 | N | Y |
| 11 | 125.2* | 91 | N | Y |
| 12 | 125.3 | 91 | N | Y |
| 13 | 125.4* | 91 | N | Y |
| 14 | 125.6 | 91 | N | Y |
| 15 | 122.1 | 83 | N | N |
| 16 | 122.2 | 83 | Y | Y |
| 17 | 122.3 | 83 | N | Y |
| 18 | 122.4 | 83 | N | Y |
| 19 | 122.5 | 83 | Y | Y |
| 20 | 122.6 | 83 | N | Y |
| 21 | 96.1 | 71 | N | N |
| 22 | 96.4 | 71 | N | N |
| 23 | 97.1 | 67 | N | Y |
| 24 | 97.2 | 67 | Y | Y |
| 25 | 130.1 | 63 | Y | Y |
| 26 | 130.2 | 63 | Y | N |
| 27 | 130.3 | 63 | Y | Y |
| 28 | 130.4 | 63 | Y | N |
| 29 | 130.5 | 63 | Y | Y |
| 30 | 130.6 | 63 | Y | N |
| 31 | 107.1 | 59 | Y | Y |
| 32 | 107.2 | 59 | Y | Y |
| 33 | 107.3 | 59 | Y | Y |
| 34 | 107.4 | 59 | N | Y |
| 35 | 107.8 | 59 | Y | Y |
| 36 | 107.9 | 59 | N | Y |
| 37 | 70.1 | 56 | N | Y |
| 38 | 70.2 | 56 | N | Y |
| 39 | 70.3 | 56 | N | ND |
| 40 | 70.4 | 56 | N | Y |
| 41 | 70.5 | 56 | Y | Y |
| 42 | 152.2 | 51 | N | ND |

**B**

|  | **Low percent ebony line number** | **% *e*** | **lbk-1** | **lbk-2** |
| --- | --- | --- | --- | --- |
| 1 | 119.1 | 44 | N | Y |
| 2 | 119.4 | 44 | N | Y |
| 3 | 119.5 | 44 | N | Y |
| 4 | 144.1 | 44 | N | N |
| 5 | 144.2 | 44 | N | ND |
| 6 | 144.4 | 44 | N | N |
| 7 | 144.5 | 44 | N | Y |
| 8 | 144.6 | 44 | N | N |
| 9 | 144.9 | 44 | N | Y |
| 10 | 144.1 | 44 | N | N |
| 11 | 149.1 | 43 | Y | Y |
| 12 | 149.2 | 43 | N | N |
| 13 | 149.3 | 43 | N | Y |
| 14 | 91.1 | 42 | N | Y |
| 15 | 91.2 | 42 | Y | Y |
| 16 | 91.3 | 42 | N | Y |
| 17 | 91.4 | 42 | N | Y |
| 18 | 82.1* | 35 | N | N |
| 19 | 82.3* | 35 | N | N |
| 20 | 139.1 | 30 | Y | Y |
| 21 | 139.2 | 30 | N | Y |
| 22 | 139.3 | 30 | N | Y |
| 23 | 139.4 | 30 | Y | N |
| 24 | 139.5 | 30 | N | N |
| 25 | 139.6 | 30 | N | Y |
| 26 | 139.7 | 30 | N | N |
| 27 | 63.1 | 28 | Y | Y |
| 28 | 63.2 | 28 | N | N |
| 29 | 63.3 | 28 | Y | N |
| 30 | 63.4 | 28 | N | N |
| 31 | 63.5 | 28 | N | ND |
| 32 | 63.6 | 28 | Y | Y |
| 33 | 63.7 | 28 | Y | Y |
| 34 | 81.3 | 23 | N | ND |
| 35 | 129.2 | 17 | N | N |
| 36 | 129.3 | 17 | N | N |
| 37 | 129.4 | 17 | N | N |
| 38 | 128.1 | 16 | Y | Y |
| 39 | 128.2 | 16 | N | N |
| 40 | 128.3 | 16 | Y | Y |
| 41 | 150.1 | 14 | Y | Y |
| 42 | 150.2 | 14 | N | Y |
| 43 | 150.4 | 14 | Y | Y |
| 44 | 148.2 | 13 | N | N |
| 45 | 147.1* | 12 | N | N |
| 46 | 151.1 | 11 | N | N |
| 47 | 151.2 | 11 | N | Y |
| 48 | 88.1 | 7 | N | Y |
| 49 | 89.1 | 7 | N | N |
| 50 | 146.1 | 6 | N | N |
| 51 | 146.2 | 6 | N | N |
| 52 | 146.3 | 6 | N | N |
| 53 | 146.4 | 6 | N | N |
| 54 | 146.5 | 6 | N | N |

**C**

|  | **Non-ebony line number** | **lbk-1** | **lbk-2** |
| --- | --- | --- | --- |
| 1 | 85.1* | N | N |
| 2 | 85.2 | N | N |
| 3 | 85.3 | N | N |
| 4 | 85.4 | N | N |
| 5 | 85.5 | N | N |
| 6 | 85.6 | N | N |
| 7 | 92.1 | N | N |
| 8 | 92.2 | N | N |
| 9 | 92.3 | N | N |
| 10 | 92.4 | N | N |
| 11 | 92.5 | N | N |
| 12 | 92.6 | N | N |
| 13 | 103.1 | N | N |
| 14 | 103.2 | N | N |
| 15 | 103.3 | N | N |
| 16 | 103.4 | N | N |
| 17 | 103.5 | N | N |
| 18 | 103.6 | N | N |
| 19 | 131.1 | N | N |
| 20 | 131.2 | N | N |
| 21 | 131.3 | N | N |
| 22 | 131.4 | N | N |
| 23 | 131.5 | N | N |
| 24 | 131.6 | N | N |

**Table S2: Mutation rates in F1 lines**. Tables list balanced *lbk* line numbers, percent *e* in F1 brood, and presence of lbk-1 and lbk-2 mutations (Y/N). **(A)** lbk-1 and lbk-2 mutation rates in jackpot lines derived from broods with >50% ebony. **(B)** lbk-1 and lbk-2 mutation rates in low ebony lines derived from broods with 1-50% ebony. **(C)** non-ebony line numbers and mutation rates. An * indicates that the line was homozygous lethal and genomic DNA was prepared from larvae. ND: not determined.
